# Identification of sphingosine kinase 1 (SphK1) as a primary target of icaritin in hepatocellular carcinoma cells

**DOI:** 10.18632/oncotarget.15205

**Published:** 2017-02-09

**Authors:** Pei-Hua Lu, Min-Bin Chen, Yuan-Yuan Liu, Mian-Hua Wu, Wen-Ting Li, Mu-Xin Wei, Chao-Ying Liu, Shu-Kui Qin

**Affiliations:** ^1^ Department of Medical Oncology, Wuxi People's Hospital Affiliated to Nanjing Medical University, Wuxi, China; ^2^ Jiangsu Collaborative Innovation Center of Traditional Chinese Medicine (TCM) Prevention and Treatment of Tumor of Nanjing University of Chinese Medicine, Nanjing, China; ^3^ Department of Oncology, Kunshan First People's Hospital Affiliated to Jiangsu University, Kunshan, China; ^4^ Department of Traditional Chinese Medicine, First Affiliated Hospital of Nanjing Medical University, Nanjing, China; ^5^ People's Liberation Army Cancer Center, 81st Hospital of People's Liberation Army, Nanjing, China

**Keywords:** hepatocellular carcinoma (HCC), icaritin, sphingosine kinase 1 (SphK1), ceramide

## Abstract

Hepatocellular carcinoma (HCC) is a highly aggressive neoplasm. We aim to explore the anti-HCC activity by a natural prenylflavonoid icaritin. Icaritin was cytotoxic and pro-apoptotic when added to established (HepG2, KYN-2 and Huh-7 lines) and primary human HCC cells. At the signaling level, icaritin inhibited sphingosine kinase 1 (SphK1) activity in HCC cells, which led to pro-apoptotic ceramide production and JNK1 activation. SphK1 inhibition or silence (by shRNA/microRNA) mimicked icaritin-mediated cytotoxicity, and almost nullified icaritin's activity in HepG2 cells. Reversely, exogenous over-expression of SphK1 sensitized icaritin-induced HepG2 cell apoptosis. *In vivo*, oral administration of icaritin dramatically inhibited HepG2 xenograft growth in SCID mice. Further, SphK1 activity in icaritin-treated tumors was largely inhibited. In summary, icaritin exerts potent anti-HCC activity *in vitro* and *in vivo*. SphK1 inhibition could be the primary mechanism of its actions in HCC cells.

## INTRODUCTION

Hepatocellular carcinoma (HCC) is a major health problem worldwide, particularly in China and other Eastern countries [1, 2]. HCC's incidence has been rising at an alarming level [1, 2]. Over the past decades, clinical treatments for HCC have achieved significant progresses, yet surgical resection remains to be the only curative therapy [1, 2]. Many HCC patients are diagnosed at advanced stages when surgery is no longer applicable [1, 2]. Further, a number of early-stage HCC patients will develop cancer recurrences or metastasis following hepatectomy [1, 2]. Significantly, HCC shows only weak response to the traditional chemotherapeutic drugs, possibly due to its high level of pre-existing or acquired resistances [1–3]. Molecule-targeted therapy is being tested in preclinical and clinical HCC studies [4, 5]. Thus far, sorafenib is the only agent approved for the systemic treatment of HCC [6, 7]. Thus, there is an urgent need to explore other novel anti-HCC agents [4, 5].

One of these agents is icaritin (IC-162), which is a hydrolytic product of icariin from the Traditional Chinese Herbal medicine *Epimedium* [8]. Studies have shown that icaritin could exert many pharmacological and biological activities, including induction of differentiation of various cells [9], prevention of steroid-associated osteonecrosis [10], and protection of neuronal cells [11]. The anti-tumor activity of icaritin has been tested in recent years. For example, it has been shown that icaritin could inhibit growth of prostate cancer PC-3 cells [12]. Further, icaritin inhibited breast cancer cell growth through activation of ERK signaling [13]. The activity of icaritin in HCC was also tested. For example, He *et al*, showed that icaritin induced HepG2 HCC cell apoptosis via activation of JNK1 signaling [14]. Further, Sun *et al*, showed that icaritin reversed multidrug resistance of HepG2 cells through downregulating MDR1 and P-glycoprotein [15]. However, the underlying signaling mechanisms, or the primary targets of icaritin, are still elusive. Further, to our best knowledge, the potential *in vivo* activity of icaritin against HCC has not been extensively tested

Existing evidences have suggested that sphingolipid metabolites are key molecule in regulating a number of cancerous behaviors [16]. In which, sphingosine-1-phosphate (S1P) promotes cancer cell survival and proliferation [17]. On the other hand, ceramide and sphingosine accumulation could promote cell apoptosis and/or growth arrest [16, 18]. The key protein kinase that regulates the balance of these sphingolipid metabolites is sphingosine kinase 1 (SphK1) [19]. SphK1 catalyses the phosphorylation of ceramide or sphingosine to S1P, thus reducing pro-apoptotic ceramide/sphingosine level, while increasing pro-survival S1P level [16, 19]. SphK1 activation positively regulates cancer cell survival, proliferation, transformation, as well as apoptosis prevention and chemo-resistance [16, 19]. Reversely, inhibition, mutation or silence of SphK1 will lead to cancer cell apoptosis and tumor repression [16, 19]. Clinical studies have shown that SphK1 is often over-expressed in a number of solid tumors including HCC [16, 19]. In the current study, we show that icaritin exerts significant anti-HCC activity both *in vitro* and *in vivo* possibly through inhibiting SphK1.

## RESULTS

### Icaritin is cytotoxic and pro-apoptotic against human HCC cells

**Figure 1:**
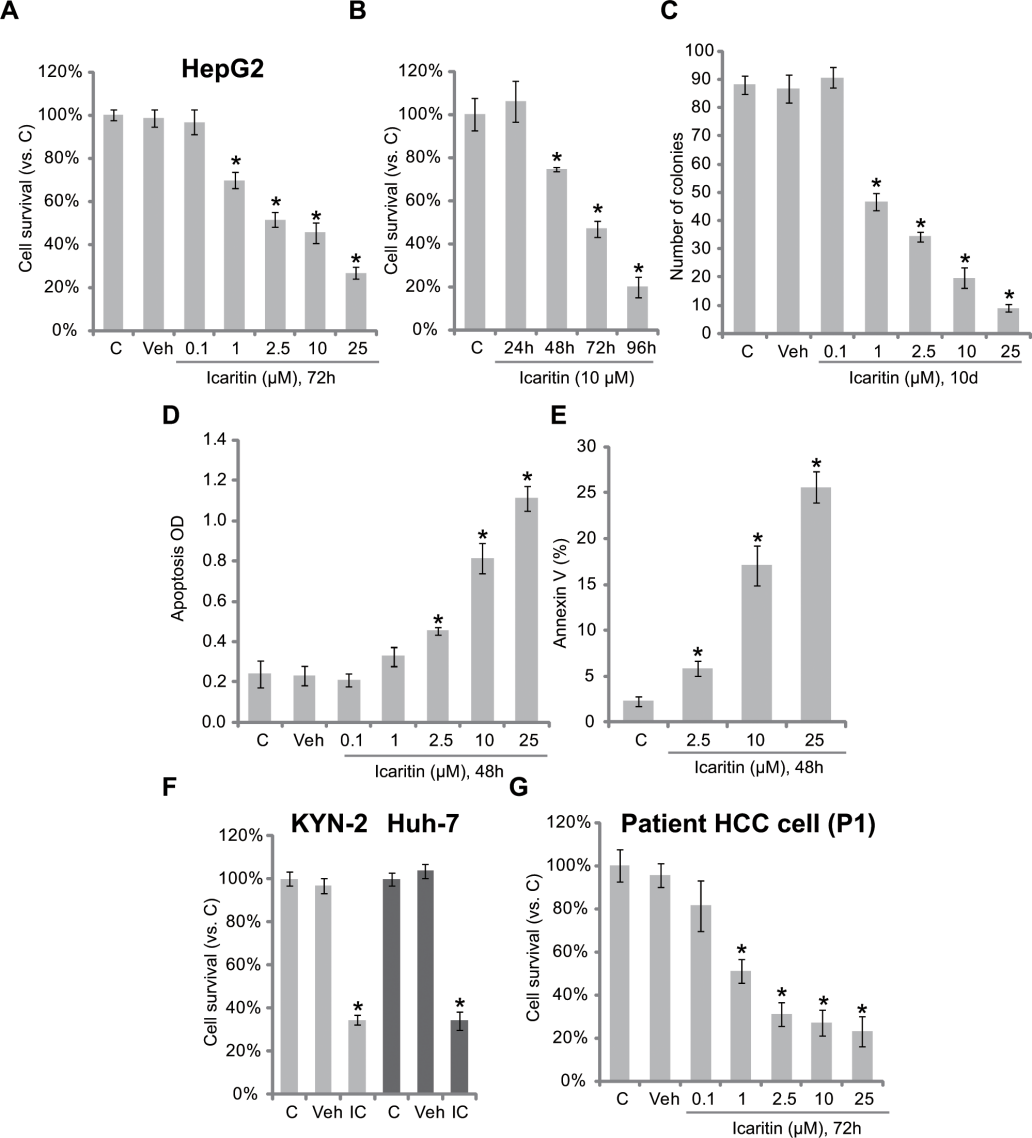
Icaritin is cytotoxic and pro-apoptotic against human HCC cells HepG2 (**A**–**E**), KYN-2 (**F**), Huh-7 (f), or primary human HCC cells (**G**, patient 1, or P1) were either left untreated (“C”, for all figures), or treated with applied concentrations of icaritin (0.1–25 μM) for indicated time, cell survival was tested by MTT assay (A, B, F and G) or clonogenicity assay (C, for HepG2 cells); HepG2 cell apoptosis was analyzed by Histone DNA ELISA assay (D) or Annexin V FACS assay (E). “IC” stands for icaritin (10 μM, 72 h) (G). Experiments in this and all following figures were repeated three times, with similar results obtained. *n* =5 for each repeat (Same for all figures). *p < 0.05 vs. group “C”. “Veh” stands for 0.1% DMSO vehicle control (Same for all figures).

We here explored the potential effect of icaritin against HCC cells. As shown in Figure 1A, icaritin treatment inhibited survival of HepG2 HCC cells in a dose-dependent manner. Icaritin was highly effective, with an IC-50 less than 5 μM (Figure 1A). Further, as shown in Figure 1B, the activity of icaritin was also time-dependent. It took at least 48 hours for icaritin (10 μM) to exert a significant anti-survival effect (Figure 1B). Colony formation in icaritin-treated HepG2 cells was also inhibited (Figure 1C). The potential effect of icaritin on HepG2 cell apoptosis was also tested. Results from the Histone DNA ELISA assay (Figure 1D) and Annexin V FACS assay (Figure 1E) demonstrated that icaritin at 2.5–25 μM induced significant HepG2 cell apoptosis. Notably, icaritin was also cytotoxic to two other human HCC cell lines: Huh-7 and KYN-2 (Figure 1F). Further, in the primary human HCC cells (Patient-1 derived, or “P1”), icaritin (1–25 μM) also decreased cell viability (Figure 1G). The experiments were also repeated in primary cancer cells derived from two other HCC patients (Patient-2/3 derived, or “P2/3”), and similar results were obtained (Supplementary Figure S1A). Note that icaritin exerted similar pro-apoptotic activity in primary (Supplementary Figure S1B) and Huh-7/KYN-2 (Supplementary Figure S1C) HCC cells. Together, these results demonstrate that icaritin is cytotoxic and pro-apoptotic against human HCC cells.

### Icaritin inhibits SphK1 activity, but increases cellular ceramide production in HCC cells

**Figure 2:**
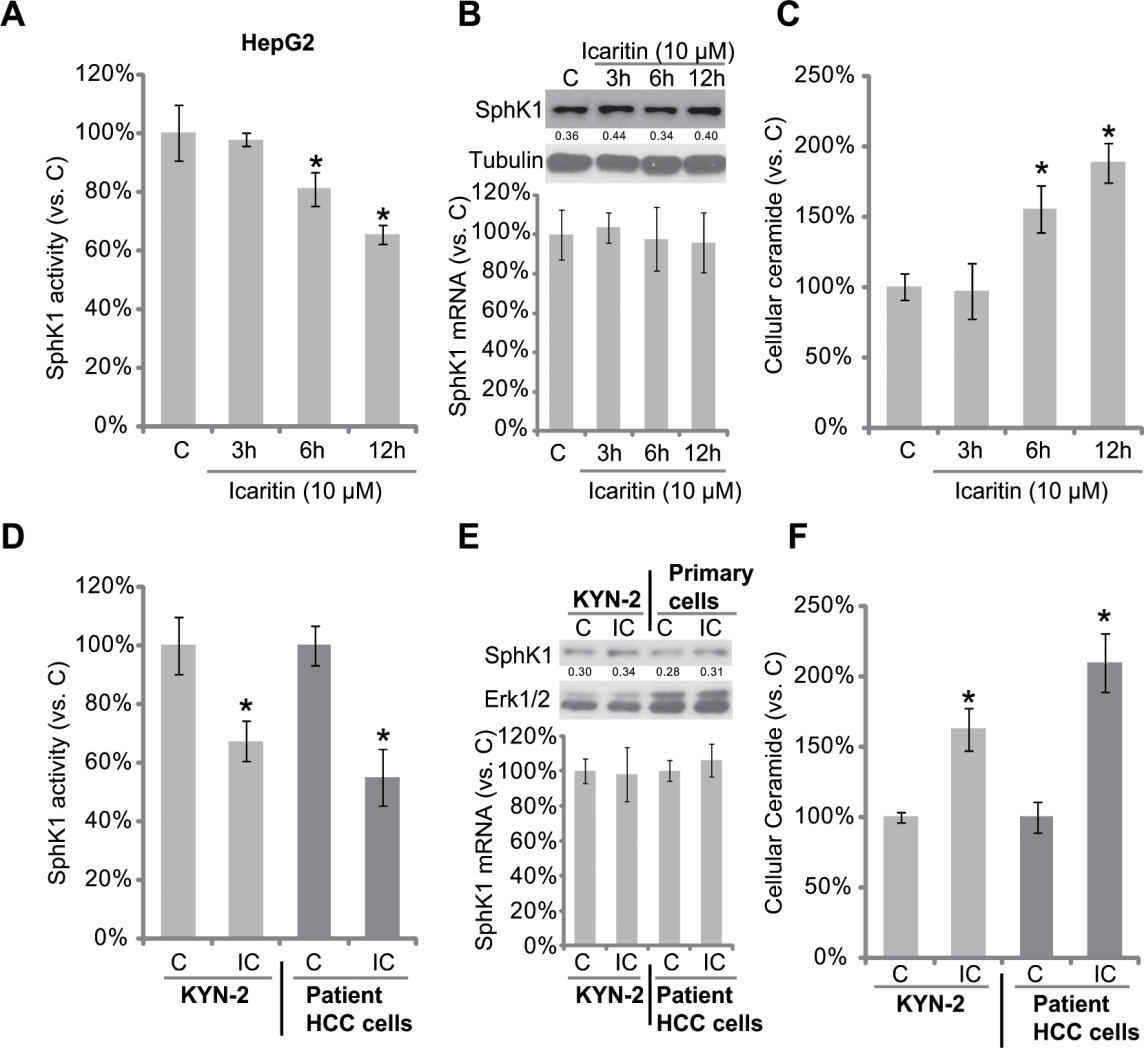
Icaritin inhibits SphK1 activity, but increases cellular ceramide production in HCC cells HepG2 (**A**–**C**), KYN-2 (**D**–**F**), or primary human HCC cells (D–F) were either left untreated, or stimulated with icaritin (10 μM) for indicated time, SphK1 activity (A and D) and cellular ceramide content (C and F) were analyzed, their levels were normalized to “C” group; SphK1 protein and mRNA expressions were analyzed by Western blot assay and real-time PCR assay, respectively (B and E). “IC” stands for icaritin (10 μM, 12 h) (D–F). *p < 0.05 vs. group “C”.

Next, the possible involvement of SphK1 in icaritin-mediated anti-HCC activity was tested. Thus, we tested the potential effect of icaritin on SphK1 activity in HCC cells. As shown in Figure 2A, icaritin treatment significantly reduced SphK1 activity in HepG2 cells. Importantly, SphK1 protein or mRNA expression was not affected by the same icaritin treatment (Figure 2B). On the other hand, the level of intracellular ceramide was increased in icaritin-treated HepG2 cells (Figure 2C). Similarly in KYN-2 cells and primary human HCC cells, the SphK1 activity, but not SphK1 expression, was decreased following icaritin treatment (Figure 2D and 2E). Consequently, the cellular ceramide level in these cells was increased (Figure 2F). Together, icaritin inhibits SphK1 activity, but increases cellular ceramide production in HCC cells.

### Ceramide production is involved in icaritin-induced JNK1 activation and HCC cell apoptosis

**Figure 3:**
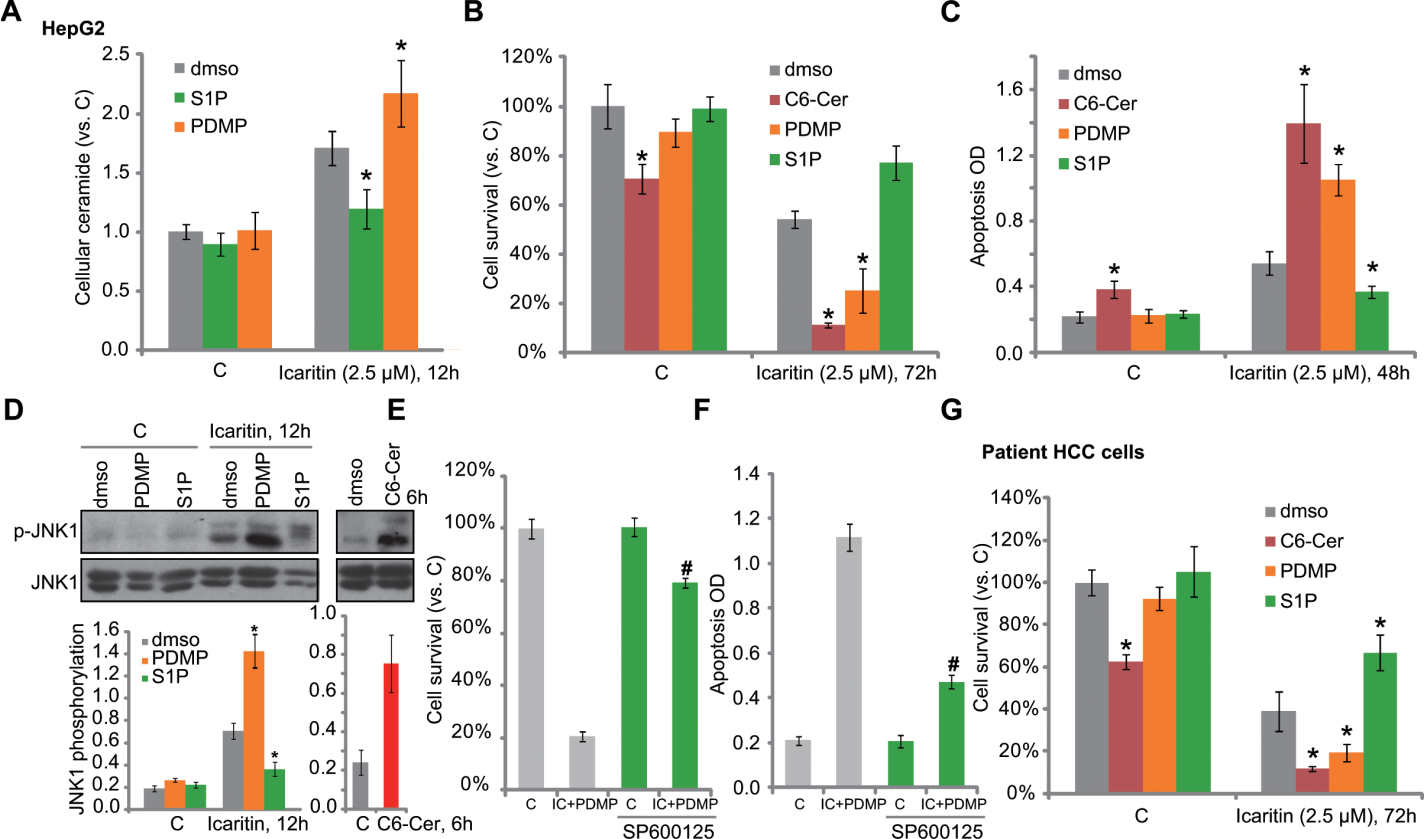
Ceramide production is involved in icaritin-induced JNK1 activation and HCC cell apoptosis HepG2 (**A**–**D**),or primary human HCC cells (**G**) were pre-treated for 1 hour with S1P (10 μM), PDMP (25 μM) or C6 ceramide (“C6-Cer”, 25 μM), followed by icaritin (2.5 μM) treatment for applied time, cellular ceramide level was analyzed (A, for HepG2 cells); Cell survival was tested by MTT assay (B and G); Cell apoptosis was analyzed by Histone DNA ELISA assay (C, for HepG2 cells), and JNK1 activation was tested by Western blot assay (D, for HepG2 cells). HepG2 cells were pre-added with the JNK inhibitor SP600125 (10 μM) for 1hour, followed by PDMP (25 μM) plus icaritin (2.5 μM) co-treatment, cell survival was tested by MTT assay (72 hours, **E**); Cell apoptosis was tested by Histone-DNA ELISA assay (48 hours, **F**). JNK1 phosphorylation (vs. regular JNK1) was quantified (D). “dmso” stands for 0.1% of DMSO. *p < 0.05 vs. group of “dmso” (A– D, **G**). ^#^*p* < 0.05 vs. group without SP600125 (E and F).

To study the potential effect of ceramide in icaritin-mediated cytotoxicity in HCC cells, pharmacological strategies were applied. PDMP is a ceramide glucosylation inhibitor [20, 21]. We showed that PDMP facilitated icaritin-induced ceramide production in HepG2 cells (Figure 3A). As a result, icaritin-induced HepG2 viability reduction (tested by MTT assay, Figure 3B) and apoptosis (tested by Histone DNA ELISA assay, Figure 3C) were both augmented with co-treatment of PDMP. On the other hand, S1P, which inhibited icaritin-induced ceramide production (Figure 3A), also attenuated subsequent HepG2 cell death and apoptosis (Figure 3B and 3C). Meanwhile, C6 ceramide, a short-chain cell-permeable ceramide [22], first mimicked icaritin's cytotoxicity (Figure 3B and 3C). It also enhanced HepG2 cell lethality by icaritin (Figure 3B and 3C).

Recent studies have shown that icaritin activates JNK1 signaling to promote cancer cell apoptosis [14, 23]. Here we found that JNK1 activation by icaritin was enhanced by PDMP, but was inhibited by S1P (Figure 3D, left). These results indicate that JNK1 activation by icaritin in HepG2 cells could also be downstream of ceramide production. In fact, treatment of C6 ceramide alone also induced JNK1 activation in HepG2 cells (Figure 3D, right). Remarkably, icaritin plus PMDP-mediated HepG2 cytotoxicity was largely inhibited by the JNK inhibitor SP600125 (Figure 3E and 3F). In primary human HCC cells, icaritin-mediated activity was again inhibited by S1P, but was aggravated by PDMP or C6 ceramide (Figure 3G). Based on these results, we propose that ceramide production is involved in icaritin-induced JNK1 activation and HCC cell death.

### SphK1 is the primary target of icaritin

**Figure 4:**
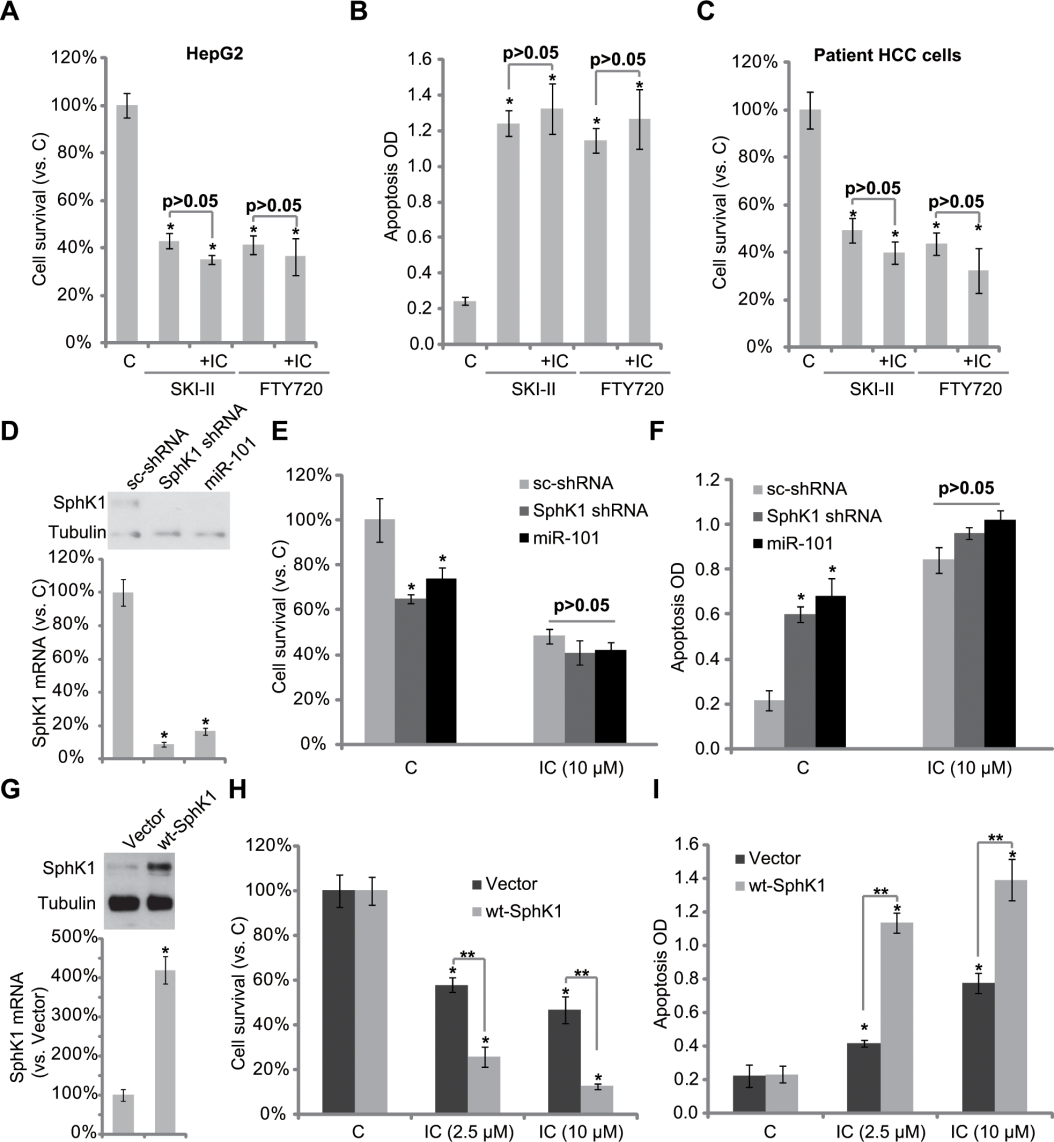
SphK1 is the primary target of icaritin in HCC cells HepG2 cells (**A** and **B**) or primary human HCC cells (**C**) were treated with icaritin (10 μM), or plus the SphK1 inhibitor SKI-II (10 μM)/FTY720 (10 μM), cell survival was tested by MTT assay (A and C, 72 hours); Cell apoptosis was tested by Histone DNA ELISA assay (B, 48 hours). Stable HepG2 cells expressing SphK1-shRNA, scramble control shRNA (“sc- shRNA”) or miR-101 as well as wt-SphK1, or the empty vector (pSuper-puro, “Vector”), were subjected to Western blot assay or real-time PCR assay to test mRNA and protein expressions of SphK1 (**D** and **G**). Above cells were also treated with applied concentrations of icaritin, cell survival (**E** and **H**, 72 hours) and apoptosis (F and I, 48 house) were tested. “IC” stands for icaritin (A–C, H and I). *p < 0.05 vs. group “C”, “sc-shRNA”, or “Vector”. **p < 0.05 (H and I).

Above results have shown that icaritin inhibited SphK1 activity, leading to pro-apoptotic ceramide production and HCC cell apoptosis. Next, we want to know if SphK1 is the primary target of icaritin. As demonstrated, two SphK1 inhibitors, SKI-II [24] and FTY720 [25], similarly induced HepG2 cell viability reduction and apoptosis (Figure 4A and 4B). Importantly, icaritin-mediated cytotoxicity in HepG2 cells was almost nullified in the presence of the two SphK1 inhibitors (Figure 4A and 4B). In another words, icaritin could no longer further inhibit HepG2 cells when SphK1 was pre-inhibited (Figure 4A and 4B). Similar results were also obtained in primary human HCC cells (P1, Figure 4C).

To exclude the off-target effects by the SphK1 inhibitors, shRNA and miRNA methods [26] were applied to knockdown SphK1 in HepG2 cells. In line with our previous findings [26], we showed that SphK1 was dramatically downregulated in HepG2 cells by SphK1 shRNA or over-expression of miRNA-101 (Figure 4D). shRNA- or miRNA-101-mediated SphK1 downregulation also induced HepG2 cell viability reduction and apoptosis (Figure 4E and 4F). Significantly, icaritin was unable to further exert cytotoxic effect in SphK1-silenced HepG2 cells (Figure 4E and 4F). These results again confirm that SphK1 is the primary target of icaritin.

To further support our hypothesis, SphK1 was over-expressed in HepG2 cells. Western blot assay and RT-PCR assay results (Figure 4G) confirmed SphK1 over-expression in the stable HepG2 cells. Notably, icaritin-mediated cytotoxicity (Figure 4H) and apoptosis (Figure 4I) were dramatically enhanced in SphK1-over-expressed HepG2 cells (Figure 4H and 4I). Similar results were also seen in two other HCC cell lines (Huh-7 and KYN-2) (Supplementary Figure S2A and S2B). On the other hand, L02 normal hepatocytes, showing extremely low level of SphK1 expression (Supplementary Figure S2C), were not killed by icaritin treatment (Supplementary Figure S2C). Together, these results further indicate that SphK1 should be the primary target of icaritin at least in HCC cells.

### Icaritin inhibits HepG2 xenograft growth in SCID mice

**Figure 5:**
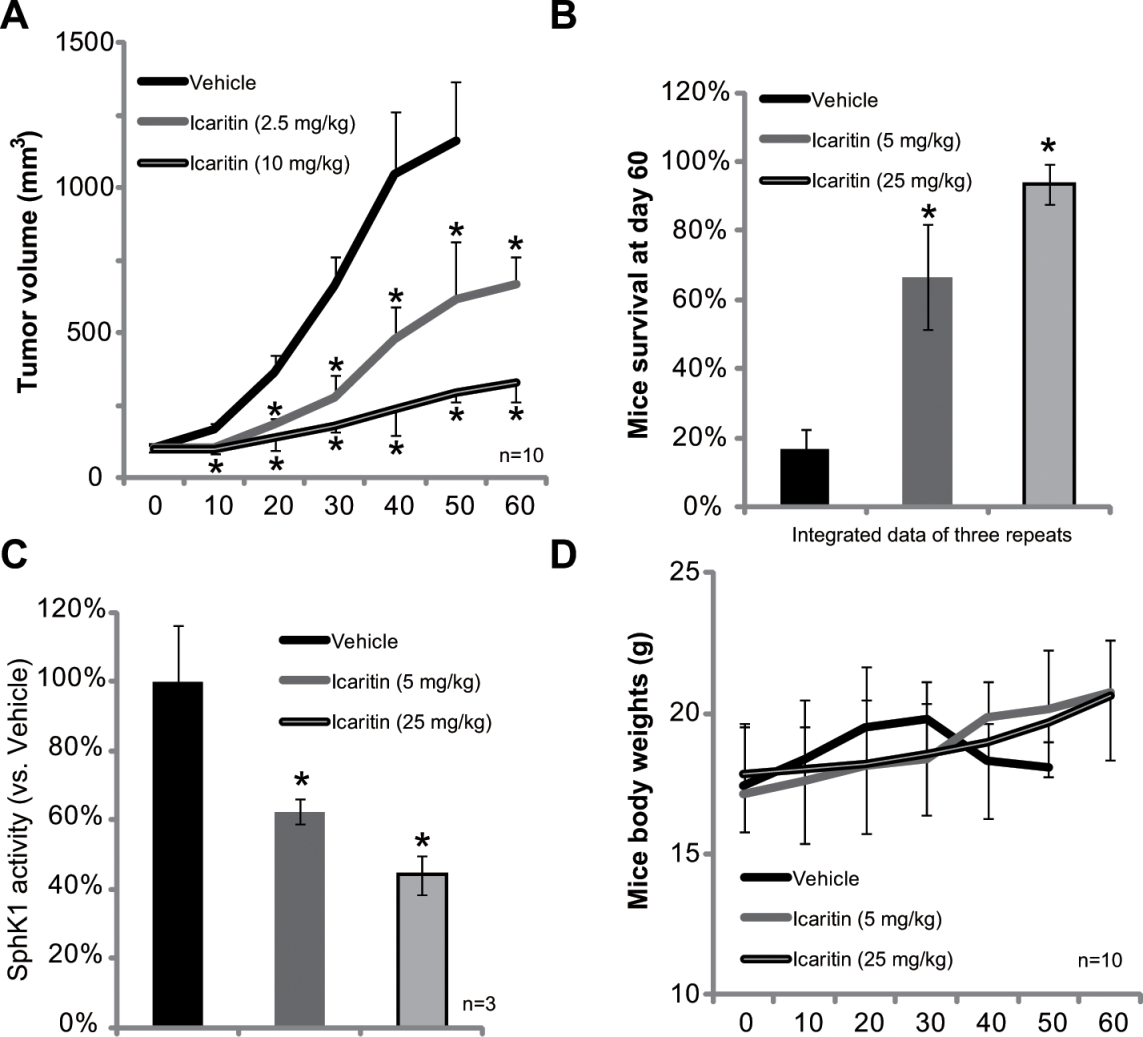
Icaritin inhibits HepG2 xenograft growth in mice The growth curve of HepG2 xenografts in SCID mice administrated with icaritin (2.5/10 mg/kg, gavage, daily) or Vehicle was shown (**A**, *n* = 10); Mice survival at day-60 was also presented (**B**, integrated data of three repeats). At the termination of experiments, HepG2 xenografts (three mice per group) were isolated through surgery, and SphK1 activity in the xenograft tissues were tested (**C**). Mice body weights were recorded (**D**, *n* = 10).*p < 0.05 vs. Vehicle group.

At last, we tested the *in vivo* activity of icaritin in the severe combined immuno-deficient (SCID) mice xenograft model. Tumor growth curve results in Figure 5A demonstrated that icaritin oral administration remarkably inhibited growth of HepG2 xenografts. The tumors in icaritin-administrated mice were much smaller than that of vehicle-treated mice (Figure 5A). Icaritin administration also significantly improved mice survival (Figure 5B). After 60 days, over 80% of the tumor bearing SCID mice with vehicle administration were already dead. On the other hand, mice with icaritin treatment were mostly alive (Figure 5B). The effect of icaritin *in vivo* was again dose-dependent (Figure 5A and 5B). Importantly, the SphK1 activity in icaritin-treated xenografts was also significantly lower than that of vehicle-treated mice (Figure 5C). The mice body weights were not affected by the icaritin treatment (Figure 5D). Also, we didn't observe any sign of toxicities (*i.e*. vomiting, diarrhea and fever) in these tested mice during the experimental duration. Together, these results show that icaritin inhibits SphK1 activity and HepG2 tumor growth *in vivo*.

## DISCUSSIONS

Groups including ours [26] have confirmed the important role of SphK1 in promoting cancer cell progression [16, 19]. Activation of SphK1 is important for cell survival, proliferation, transformation, as well as metastasis and chemo-resistance [16, 19]. Meanwhile, SphK1 is over-expressed in multiple solid tumors, which is often associated with poor prognosis [16, 19]. SphK1 inhibitors, alone or in combination with conventional anti-cancer agents, have demonstrated promising anti-tumor results [16, 19]. Recent studies have explored the potential role of SphK1 in HCC. For example, Shi *et al*., showed that SphK1 is over-expressed in multiple human HCC tissues [27]. Further, patients with SphK1 over-expression are often with poor prognosis [27]. *In vitro*, existing evidence demonstrated that SphK1 participates in HCC cell migration and invasion [28]. Zhang *et al*., showed that SphK1 inhibitors suppressed HCC cell proliferation and invasion possibly through inhibition of SphK1-NFκB signaling [29]. All these studies pointed out that SphK1 could be a valuable oncotarget for HCC. Here, we showed that icaritin exerts anti-HCC activity possibly through inhibiting SphK1.

We provided evidence to show that SphK1 is the primary target of icaritin in HCC cells. Icaritin dramatically inhibited SphK1 activity in HCC cells. As a consequence, the intracellular ceramide level was increased, which mediated subsequent HCC cell apoptosis. Icaritin-induced ceramide production and HCC cytotoxicity were enhanced by PDMP, the ceramide glucosylation inhibitor, but were remarkably attenuated by S1P, the latter inhibited ceramide production. Further, pharmacological inhibition or genetic silence of SphK1 mimicked icaritin's cytotoxicity against HCC cells, and almost completely nullified icaritin-exerted activity in HCC cells. On the other hand, exogenous over-expression of SphK1 dramatically sensitized icaritin-mediated cytotoxicity. *In vivo*, icaritin oral administration remarkably inhibited HepG2 xenograft growth in SCID mice. Further, SphK1 activity was also decreased in icaritin-treated xenograft tissues. Based on these results, we propose that icaritin exerts significant anti-HCC activity possibly through inhibiting SphK1.

A recent study by Zhao *et al*, [30] showed that pSTAT3 level was significantly increased in HCC cells. Further, icaritin-mediated anti-HCC cell activity was associated with STAT3 inhibition [30]. Here we found that SphK1 silence (by shRNA or miR-101) significantly inhibited STAT3 activation (pSTAT3 at Y705) in HepG2 cells (Supplementary Figure S3A). On the other hand, SphK1 over-expression increased pSTAT3 level (Supplementary Figure S3B). Therefore, SphK1 could be the upstream signal of STAT3 in HCC cells. As a matter of fact, Liang *et al*., has already shown that SphK1 activation leads to S1P production, which activate STAT3 [31]. Therefore, we propose that icaritin inhibits SphK1 activation, causing S1P depletion and downstream STAT3 inhibition, which could be responsible for HCC cell apoptosis [30]. The detailed mechanisms warrant further investigations.

Recent studies have demonstrated the ability of icaritin to activate pro-apoptotic JNK1 in HepG2 cells [14]. SP600125, a pharmacological inhibitor of JNK1, was shown to repress HepG2 cell apoptosis by icaritin [14]. Here we suggest that JNK1 activation could be the downstream effector of SphK1-cearmaide signaling in icaritin-treated cells. PDMP, which facilitated ceramide production by icaritin, also enhanced following JNK1 activation. Significantly, SP600125 dramatically attenuated PDMP plus icaritin-induced HepG2 cytotoxicity. On the other hand, S1P attenuated ceramide production and subsequent JNK1 activation by icaritin. These results are not surprising, as ceramide is known to activate JNK-dependent apoptosis [32, 33]. Inhibition, silence or mutation of JNK1 could block cell apoptosis by ceramide [32, 33]. Based on these results, we propose that icaritin inhibits SphK1 to increase ceramide accumulation, which activates JNK1 signaling to promote HCC cell apoptosis.

The chemo-resistant nature of HCC and the ineffectiveness of the current chemo-agents require more effective anti-HCC agents [2, 4, 5]. The sorafenib-based chemotherapy has only demonstrated minimal benefits in improving HCC patients’ survival [2, 4, 5]. In the current study, we showed that icaritin was more potent than same concentration of sorafenib in inhibiting HepG2 cells (Supplementary Figure S4). Further, we found that L02 normal hepatocytes were not killed by icaritin treatment (Supplementary Figure S2C). When given *in vivo*, we and others [30] found that icaritin failed to cause any apparent toxicities in mice. Therefore, it will be interesting to further explore the potential anti-HCC activity by icaritin in clinical settings.

## CONCLUSIONS

In summary, the results of this study imply that icaritin exerts significant anti-HCC activities *in vitro* and *in vivo*, probably via inhibiting SphK1 signaling.

## MATERIALS AND METHODS

### Chemicals and reagents

Icaritin (purity 98%) was purchased from Shenogen Pharma Group (Beijing, China). Icaritin was dissolved in dimethyl sulfoxide (DMSO) for *in vitro* studies. The final concentration of DMSO in the culture media was maintained at 0.1%, which showed no cytotoxicity to HCC cells. For *in vivo* studies, icaritin was dissolved in the SX-1292 oral vehicle [1% sodium carboxymethyl cellulose, 0.5% sodium lauryl sulfate (SLS), and 0.05% antifoam; Eli Lilly, Shanghai, China] and administered by gastric lavage. D-threo-1-phenyl-2-decanoylamino-3-morpholino-1-propanol (PDMP), FTY720 and SP600125 were obtained from Sigma (St. Louis, MO); SKI-II (4-[[4-(4-Chlorophenyl)-2-thiazolyl]amino]phenol) and sorafenib were purchased from Tocris Bioscience (Ellisville, Mo). Sphingosine 1-phosphate (S1P) and C6 ceramide were obtained from Avanti Polar Lipids, Inc. (Alabaster, AL). All antibodies utilized in this study were purchased from Cell Signaling Technology (Beverly, MA).

### Cell lines

Established HCC cells (HepG2, KYN-2 and Huh-7 lines), and L02 normal hepatocytes, purchased from the Cell Bank of Shanghai Biological Institute (Shanghai, China), were grown in DMEM/RPMI medium supplemented with 5–10% fetal bovine serum (FBS, Gibco, Shanghai, China), penicillin (100 units/mL), and streptomycin (100 μg/mL) in an atmosphere of 5% CO_2_. L02 normal hepatocytes were cultured as described [30].

### Patient-derived HCC cells

Surgery-isolated HCC tissues from informed-consent patients (three male patients, 45–55 years old. P1/P2/P3) were thoroughly washed in DMEM and 1 mM DTT (Sigma). Tissues were subjected to digestion for 1 hour. Single-cell suspensions were then pelleted and washed, before re-suspending the cells in culture medium (DMEM, 20%-FBS, 2 mM glutamine, 1 mM pyruvate, 10 mM HEPES, 100 units/mL penicillin/streptomycin, 0.1 mg/mL gentamicin, and 2 g/liter fungizone) [34–36]. All investigations with clinical samples were in accordance with the principles expressed in the Declaration of Helsinki, and was approved by the Institutional Review Board (IRB) and Ethics Board of all authors’ institutions.

### Methyl thiazol tetrazolium (MTT) assay

Cell viability was assessed using the MTT assay as described [37–39].

### Colony formation assay

As previously reported [26], HCC cell were suspended in DMEM with 0.35% agar, 10 % FBS, which was then added on the top of a culture dish. After 10 days of incubation, the number of colonies were fixed, stained and manually counted.

### Apoptosis assay

After treatment, HCC cell apoptosis was analyzed by Annexin V FACS assay. The percentage of Annexin V was utilized as a quantitative measurement of cell apoptosis.

### Histone-DNA enzyme-linked immunosorbent assay (ELISA) assay

Cell apoptosis was quantified by Histone-DNA ELISA PLUS kit (Roche Applied Science, Shanghai, China) according to the manufacturer's protocol [37–39].

### Western blots

Western blots were performed as previously described [37–39]. All blots in this study were subjected to different exposures: from 10 seconds to 10 minutes. Blot intensity was quantified by ImageJ software (NIH) after normalization to corresponding loading controls.

### The SphK1 activity assay

The SphK1 activity was analyzed with the help from Dr. Gu's group [40]. Briefly, cell lysates (100 μg/sample) were incubated with 25 μM D-erythrosphingosine dissolved in 0.1% Triton X-100, 2 mM ATP, and [^γ-32^P] ATP for 30 min at 37°C. The reaction was terminated through adding 20 μL of HCl, plus 800 μL of chloroform/methanol/HCl (100:200:1, v/v). After vortex, 250 μL of chloroform and 250 μL of KCl were added, and phases were separated by centrifugation. The organic layer was dried and resuspended in chloroform/methanol/HCl 37% (100:100:0.2, v/v). Lipids were resolved on silica TLC plates in 1-butanol/acetic acid/water (3:1:1, v/v). Labeled S1P spots were visualized by autoradiography and quantified by scraping and counting in a scintillation counter. The SphK1 activity was valued as pmol/hour/g protein.

### Cellular ceramide assay

The ceramide level was analyzed by the same method described in [41], and was valued as fmol by nmol of phospholipid (PL).

### Constructs and transfection

As reported, the miR-101 precursor [26] was sub-cloned into pSuper-puro-GFP vector to generate miR-101 expression construct. The SphK1-shRNA-puromycin construct was purchased from Santa Cruz Biotech (sc-44114-SH, Santa Cruz, CA). For transfection, HepG2 cells were seeded onto six-well plate at 50–70% confluence. HepG2 cells were transfected via the Lipofectamine 2000 reagents (Invitrogen, USA). After 24 hours of incubation at 37°C, transfection medium was replaced with 2 mL of complete medium. Puromycin (5.0 μg/mL, Sigma) was added after transfection to select stable cells (6 days). miR-101 or SphK1 expression in the stable cells was always tested.

### RNA extraction and real-time PCR

Total RNA was prepared using TRIzol reagents (Invitrogen, Shanghai, China). Real time-PCR assay was performed based on the protocol as previously reported [26]. Corresponding primers were also described early [26]. After amplification, melt curve analysis was performed to analyze product melting temperature. *GAPDH* was tested as the reference gene for normalization, and the 2^−ΔΔ^C^t^ method was applied to quantify targeted-mRNA expression change within samples.

### SphK1 over-expression

A cDNA encoding full-length human SphK1 (from HepG2 cells, synthesized and verified by Genepharm, Shanghai, China) was sub-cloned into the pSUPER-puro-retro vector [42], which was then transfected into HEK-293 cells with plasmids encoding viral packaging proteins VSVG and Hit-60 (Promega) [42] using Lipofectamine 2000 (Invitrogen) reagent. Two days post-transfection, the medium containing virus particles was added to HepG2 cells, and infections were allowed to proceed for 24 hours. The vector-expressing cells were selected post infection in the presence of 5.0 μg/mL of puromycin for 10 days. Over-expression of SphK1 in the transfected cells was tested by Western blot assay and RT-PCR.

### Tumor xenografts

*M*ale severe combined immuno-deficient (SCID) mice (7–8 week-old) were implanted *s.c*. with HepG2 cells (2*10^6^ cells per mouse). When the xenografted tumors reached the average volume of 100 mm^3^, the mice were treated with icaritin (2.5/10 mg/kg, gavage, daily, for 30 day) or vehicle, with 10 mice per group. Tumor volumes were recorded every 10 days, calculated using the following formula:*π*/6 × larger diameter × (smaller diameter)^2^ [43]. Mice body weights were also recorded. After 60 days, mice survival was also recorded. All studies were performed in accordance with the standards of ethical treatment approved by the Institutional Animal Care and Use Committee (IACUC) and Association for the Assessment and Accreditation of Laboratory Animal Care (AAALAC).

### Statistical analysis

Data were presented as mean ± standard deviation(SD). Statistics were analyzed by one-way ANOVA followed by a Scheffe’ and Tukey Test by SPSS 18.0 software (SPSS Inc., Chicago, IL). Significance was chosen as *p* < 0.05. The concentrations of agents and the treatment durations were chosen based on published literatures and results from pre-experiments.

## SUPPLEMENTARY FIGURES


