# Supplementary material for: Identification of sphingosine kinase 1 (SphK1) as a primary target of icaritin in hepatocellular carcinoma cells
Source: Oncotarget. 2017 Feb 9;8(14):22800–10. doi: 10.18632/oncotarget.15205 (PMC5410263; doi:10.18632/oncotarget.15205)
Supplement: Supplementary file 1 [file oncotarget-08-22800-s001.pdf]

Identification of sphingosine kinase 1 (SphK1) as a primary target of icaritin in hepatocellular carcinoma cells

Supplementary Materials

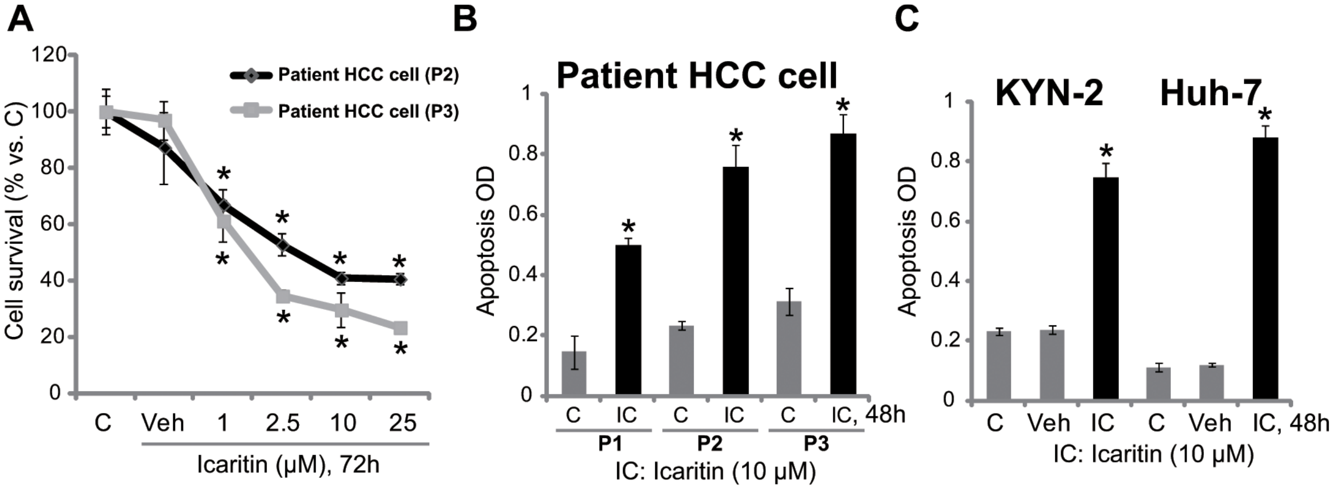

Supplementary Figure S1: Listed HCC cells were treated with icaritin for indicated time, cell survival was tested by MTT assay (A); Cell apoptosis was examined by the Histone DNA apoptosis ELISA assay (B and C). *$p < 0.05$ vs. group "C".

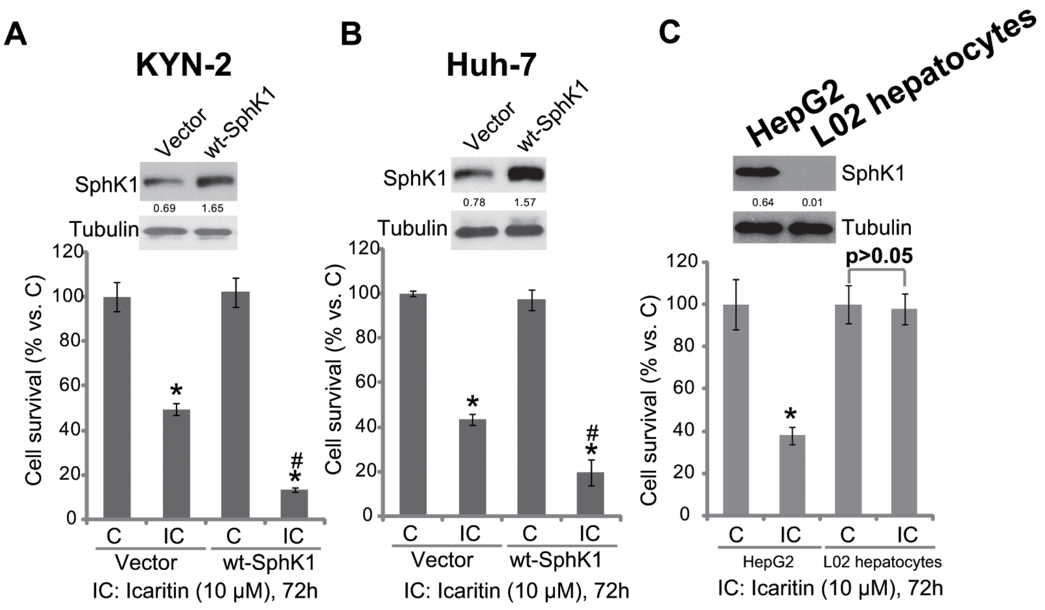

Supplementary Figure S2: Stable Huh-7 cells (A) or KYN-2 cells (B) expressing wt-SphK1, or the empty vector (pSuper-puro, "Vector"), as well as HepG2 cells (C) and L02 hepatocytes (C) were subjected to Western blot assay to test protein expression of SphK1. Above cells were also treated with icaritin, cell survival (MTT assay) were tested. SphK1 expression (vs. Tubulin) was quantified. *$p < 0.05$ vs. group "C". $^{\#}p < 0.05$ vs. group ""Vector".

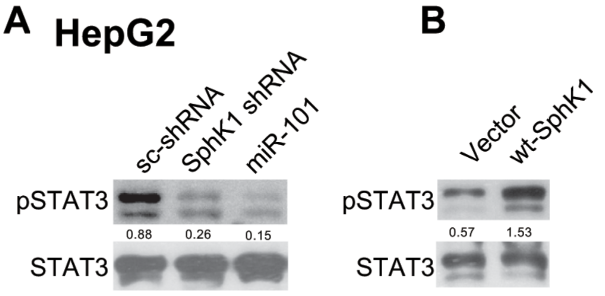

Supplementary Figure S3: Stable HepG2 cells expressing SphK1-shRNA, scramble control shRNA ("sc-shRNA") or miR-101 (A) as well as wt-SphK1, or the empty vector (pSuper-puro, "Vector") (B), were subjected to Western blot assay of phosphorylated (Y705) and regular STAT3. STAT3 phosphorylation was quantified.

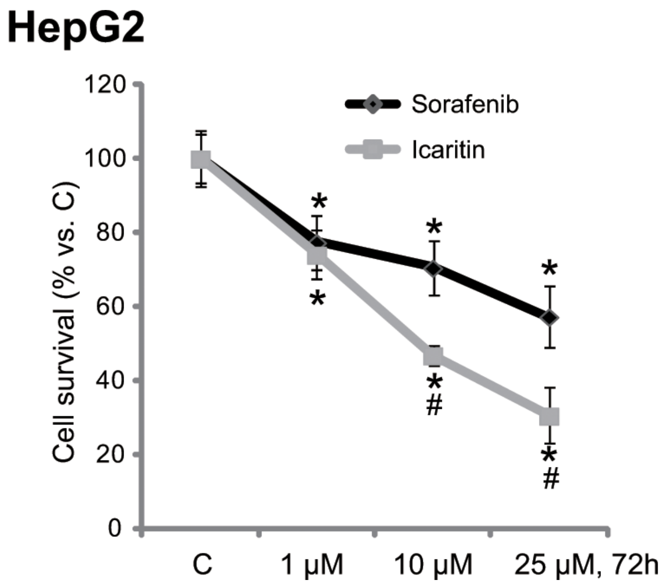

Supplementary Figure S4: HepG2 cells were treated with applied concentrations of icaritin or sorafenib for 72 hours, cell survival was tested by MTT assay. *$p < 0.05$ vs. group "C". #$p < 0.05$ vs. same concentration of icaritin.
